# American Med. Association

**Published:** 1879-03-20

**Authors:** 


### *AMERICAN MEDICAL ASSOCIATION.*

This Association is appointed to meet in this city (Atlanta) on the first Tuesday in May next. In our April issue we hope to be able to state the programme for the occasion.

It is anticipated that in addition to the usual number of delegates that a great many visiting brethren will be in attendance from the adjacent States, swelling the number in the aggregate to several hundreds.

These meetings have rarely been held in the Southern States, there being few cities able to accommodate so large a convention.

While our city is yet struggling from the ashes of war, and the members of the Profession are generally poor and cannot be expected to give costly entertainments or make extraordinary displays, yet we doubt not that the members of the Association and the visitors will be received with open arms and a cordial welcome.

